# CRISPR-Mediated Base Editing: From Precise Point Mutation to Genome-Wide Engineering in Nonmodel Microbes

**DOI:** 10.3390/biology11040571

**Published:** 2022-04-09

**Authors:** Mengyuan Li, Yi-Xin Huo, Shuyuan Guo

**Affiliations:** Key Laboratory of Molecular Medicine and Biotherapy, School of Life Science, Beijing Institute of Technology, No. 5 South Zhongguancun Street, Beijing 100081, China; mengyuaninuf@outlook.com

**Keywords:** base editing, nonmodel microbes, gene engineering, CRISPR/Cas9, in vivo evolution

## Abstract

**Simple Summary:**

Gene engineering techniques are essential for genetic characterizations and metabolic engineering. A stable and robust gene editing method can speed up the explorations of nonmodel microbes which show tremendous potential for a variety of applications. In recent years, base editors have achieved precise point mutation and multiplex gene editing in a wide range of microbes. Without causing double stranded breaks and requiring a donor DNA template, base editors are more available than CRISPR/Cas9 for those species with a low homologous recombination system. Here, we introduce the latest development and applications of base editors in nonmodel microbes. This versatile method is suitable for gene editing from precise point mutation to genome-wide engineering in nonmodel microbes and holds good promise for future development of nonmodel microbes.

**Abstract:**

Nonmodel microbes with unique and diverse metabolisms have become rising stars in synthetic biology; however, the lack of efficient gene engineering techniques still hinders their development. Recently, the use of base editors has emerged as a versatile method for gene engineering in a wide range of organisms including nonmodel microbes. This method is a fusion of impaired CRISPR/Cas9 nuclease and base deaminase, enabling the precise point mutation at the target without inducing homologous recombination. This review updates the latest advancement of base editors in microbes, including the conclusion of all microbes that have been researched by base editors, the introduction of newly developed base editors, and their applications. We provide a list that comprehensively concludes specific applications of BEs in nonmodel microbes, which play important roles in industrial, agricultural, and clinical fields. We also present some microbes in which BEs have not been fully established, in the hope that they are explored further and so that other microbial species can achieve arbitrary base conversions. The current obstacles facing BEs and solutions are put forward. Lastly, the highly efficient BEs and other developed versions for genome-wide reprogramming of cells are discussed, showing great potential for future engineering of nonmodel microbes.

## 1. Introduction

Microbial species play essential roles in the human world, which are both attractive and hateful to humans. On the one hand, they are applied widely in the agricultural, pharmaceutical, biofuel, and food industries, and so on [1,2]. On the other hand, microbial pathogens cause severe infections with the rising resistance against drugs. Since the yields and productivity of host strains are not economically competitive [3,4], and the production costs are high via industrial fermentation [3,5], it is crucial to achieve highly efficient and economical production. Previous technologies, including repeated cycles of screening for desired phenotypes through physical or chemical mutagenesis, and fermentation optimization, have improved the microbial productivity and reduced costs [6]; however, these methods are still challenging in terms of improving the yields of desired products, due to the limited mutation rates and unwanted genetic alterations. In order to increase the production of the target metabolites, synthetic biology and metabolic engineering are developed to optimize genetic and regulatory processes within cells. One of the most useful tools to learn and modify biosynthetic pathways is gene engineering techniques, which can ultimately alter microbial genotypes and phenotypes by insertions, deletions, and mutations of nucleotides [7]. Model microbes such as *Escherichia coli* (*E. coli*) and *Saccharomyces cerevisiae* (*S. cerevisiae*) have been researched and used for industrial biochemical production in the long term [8]. Despite their well-characterized metabolisms and availability of a large genetic toolbox for rapid gene modification [9], these model strains are unable to produce all desirable products to satisfy the increasing industrial needs.

In contrast, nonmodel microbes that are derived from a more complex environment and have evolved to utilize cheaper and more environmentally friendly sources of carbon than model microbes, often possess versatile physiology and metabolic capabilities that are important for the future production of biofuel, as well as chemical and novel antibiotics that may ease the problem of multi-drug resistance [8,10]. They are also able to tolerate extreme industrial processing environments, such as low pH, high salt, and high temperatures; therefore, nonmodel microbes have become one of the hot spots in metabolic engineering. However, the lack of facile gene editing tools hinders the genetic characterizations and modifications of nonmodel microbes [11]. Moreover, the understanding and mitigating drug-resistance mechanisms require the gene engineering approaches to aid in targeting and editing pathogenic microbial genomes [12].

**Figure 1:**
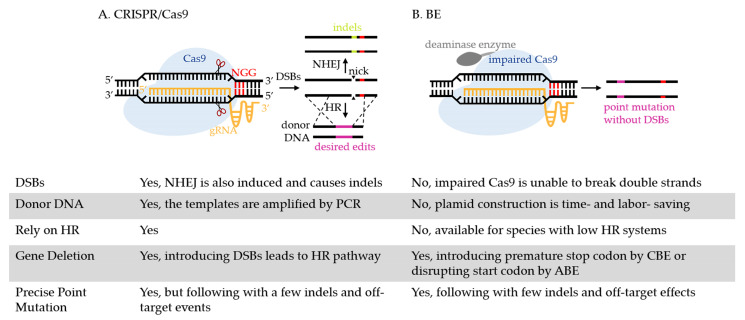
The process of gene editing using CRISPR/Cas9 and a BE. (**A**) Directed by gRNA, CRISPR/Cas9 recognizes the PAM site (5′-NGG-3′, highlighted in red) and introduces DSBs at the target. DSBs induces two cellular DNA repair systems, NHEJ and HR. Through NHEJ, a few imprecise indels are created through error-prone DNA repair. Through HR, the target is replaced by homologous donor DNA with desired edits and is therefore introduced with deletions, insertions, or substitutions of nucleotides. (**B**) The BE is also directed by gRNA to the target. At the target, deaminase enzyme achieves base conversion without causing DSBs and requiring donor DNA. CBE mediates the conversion of a C:G base pair to a T:A, which can replace four codons (CAA, CAG, CGA, TGG) with premature stop codons (TAA, TAG, TGA) to inactivate the gene. ABE mediates the base conversion from an A:T base pair to G:C, which can replace start codon ATG with GTG or ACG to disrupt the initiation of gene translation. Other than point mutation, BEs can achieve multiplex gene editing, in vivo evolution of protein and strain, metabolic engineering, etc., in various microbes, as discussed in detail below.

The advent of the CRISPR/Cas9 (CRISPR, clustered regularly interspaced short palindromic repeats/Cas9, the dual RNA-guided DNA endonuclease) opens the door to manipulate the genome of microbes that are traditionally difficult to be edited (such as nonmodel bacteria [13,14,15,16], fungi [17], protozoan parasites [18,19], etc.). As shown in Figure 1A, the guide RNA (gRNA) directs Cas9 to the targeted DNA containing the protospacer adjacent motif (PAM, 5′-NGG-3′) to introduce DNA double-stranded breaks (DSBs) [20]. This induces homologous recombination (HR) to replace the DNA sequence with homologous templates. However, the generation of DSBs is reported to be lethal to some bacteria, such as *Clostridium cellulolyticum* [21] and *E. coli* [22], and it induces nonhomologous end joining (NHEJ), which results in random insertions or deletions (indels) [23,24]. The lack of a strong HR system in some microbes, such as *Mycobacterium tuberculosis* (*M. tuberculosis*) [25] and *Yarrowia lipolytica* [26], also adds difficulties to CRISPR/Cas9 editing.

Recently, a base editor (BE), which was fusing a deaminase enzyme with an impaired CRISPR/Cas nuclease (unable to cleave DNA double strands) together [27], has emerged as an efficient template-free gene editing method. The impaired Cas nuclease generated from *Streptococcus pyogenes* Cas9 (SpCas9) can either be a catalytically dead Cas9 (dCas9 containing D10A and H840A mutations), that is completely unable to cleave DNA double strands, or a Cas9 nickase (nCas9, retaining D10A mutation) that nicks the nonedited strand [28]. In a gRNA-programmed manner, the fusion protein is recruited to the target without causing DSBs [29], where cytosine deaminase is fused with impaired Cas9 (cytosine base editor, CBE) to convert cytidine (C) to thymidine (T), and adenine deaminase is fused with impaired Cas9 (adenine base editor, ABE) to convert adenosine (A) to guanosine (G). As shown in Figure 1, BEs are more convenient and refined for precise gene editing than CRISPR/Cas9.

## 2. Advancement of BEs in Microbes

**Figure 2:**
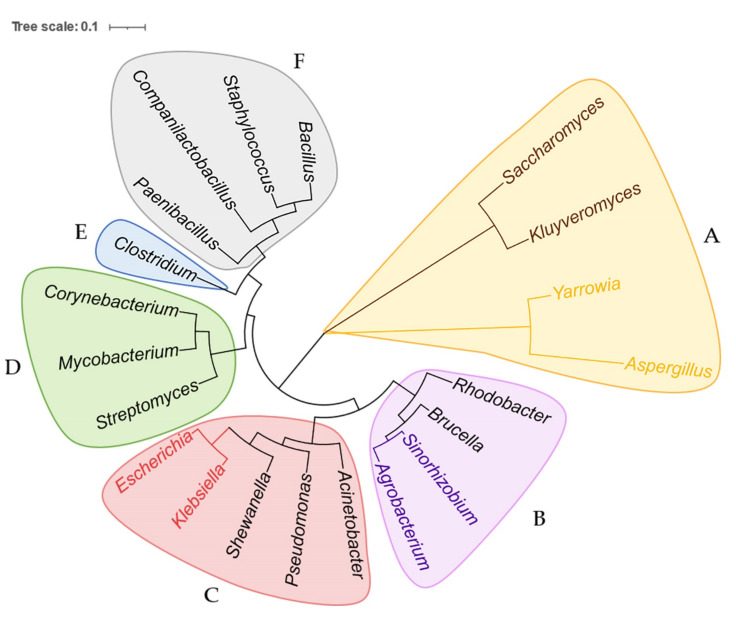
Phylogenetic tree of all microbes that have been researched by BEs, based on small-subunit rRNA sequences and constructed using the maximum-likelihood estimation. The phylogenetic tree includes 21 genera and six classes which are shadowed in different colors. (**A**) Microbes belong to the kingdom Fungi, phylum Ascomycota, and class Saccharomycetes. *Saccharomyces* and *Kluyveromyces* belong to the same family, Saccharomycetaceae, highlighted in brown. *Yarrowia* and *Aspergillus* belong to the same family, Dipodascaceae, highlighted in yellow. (**B**) Microbes belong to the kingdom Bacteria, phylum Proteobacteria, and class Alphaproteobacteria. *Rhodobacter* and *Brucella* belong to the family Rhodobacteraceae and Rhizobiaceae, respectively. *Agrobacterium* and *Sinorhizobium* belong to the same family, Brucellaceae, highlighted in purple. (**C**) Microbes belong to the kingdom Bacteria, phylum Proteobacteria, and class Gammaproteobacteria. *Pseudomonas*, *Acinetobacter*, and *Shewanella* belong to the family Pseudomonadaceae, Moraxellaceae, and Shewanellaceae, respectively. *Escherichia* and *Klebsiella* belong to the same family, Enterobacteriaceae, highlighted in pink. (**D**) Microbes belong to the kingdom Bacteria, phylum Actinobacteria, and class Actinomycetia. *Streptomyces*, *Mycobacterium*, and *Corynebacterium* belong to the family Streptomycetaceae, Corynebacteriaceae, and Mycobacteriaceae, respectively. (**E**) Genus *Clostridium* belongs to the kingdom Bacteria, phylum Firmicutes, class Clostridia, and family Clostridiaceae. (**F**) Microbes belong to kingdom Bacteria, phylum Firmicutes, and class Bacilli. *Paenibacillus*, *Companilactobacillus*, *Staphylococcus*, and *Bacillus* belong to the family Paenibacillaceae, Lactobacillaceae, Staphylococcaceae, and Bacillaceae, respectively.

Previous reviews [30,31,32] have concluded the applications of BEs in many microbes, such as model species *S. cerevisiae* [33,34,35], *E. coli* [36,37,38], *Corynebacterium glutamicum* (*C. glutamicum*) [39,40,41], *Aspergillus niger* (*A. niger*) [42], and nonmodel microbes *Psedomonas* spp. [43], *Yarrowia lipolytica* (*Y. lipolytica*) [26], *Strptomyces* spp. [44,45,46], *Clostridium beijerinckii* (*C. beijerinckii*) [47], *Rhodobacter sphaeroides* (*R. sphaeroides*) [48], and *Shewanella oneidensis* (*S. oneidensis*) [49]. However, since then, BEs have been further extended to new types and applied in new species (especially nonmodel microbes); therefore we construct a universal phylogenetic tree in Figure 2 to directly present all current microbial genera in which BEs have been established so far. The newly developed BEs that have overcome the editing limitations in microbes are further discussed below and listed in Table 1. After that, we focus on the specific applications of BEs in nonmodel microbes. The successful and failed examples of BEs in nonmodel microbes, which have rarely been concluded before, are comprehensively listed in Table 2, providing a friendly guide for future research.

### 2.1. Constructs and Mechanisms of Developed BEs

#### 2.1.1. Latest Development of CBE and ABE in Microbes

**Figure 3:**
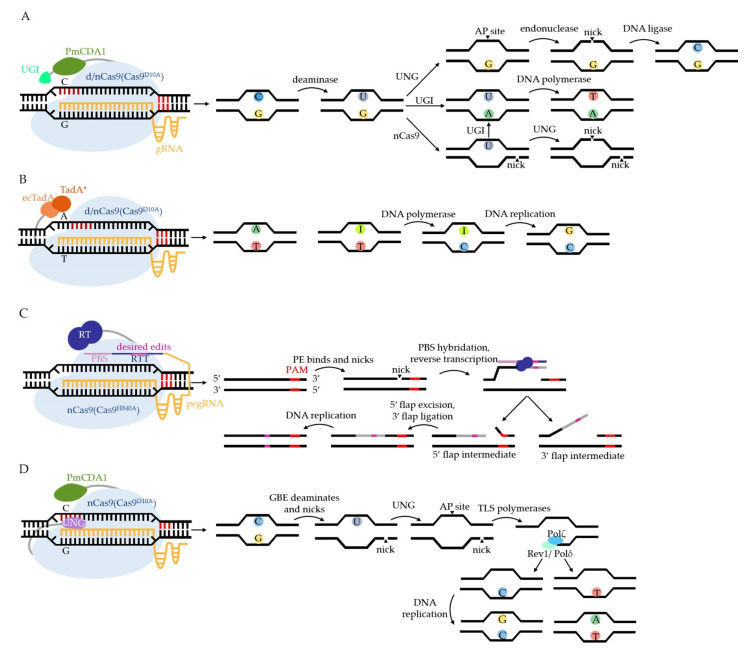
The basic components and working mechanisms of CRISPR-mediated base editing systems. The common editing windows are highlighted in dark red in the DNA sequence. (**A**) CBE consists of cytosine deaminase (PmCDA1 in (A)), impaired Cas9, gRNA, and UGI (inhibits uracil DNA glycosylase, abbreviated to UNG, to improve the efficiency of CBE but is not essential). Under the R-loop, CBE deaminates C to U. If UNG removes U, the apurinic/apyrimidinic (AP site) will soon be reversed to C. If UGI is added to CBE, U will be retained at the locus where DNA polymerase will read U as T. The C:G is successfully conversed to T:A. If dCas9 is replaced with nCas9, the nonedited strand will be nicked. Without the inhibition of UGI, U will be removed at the other strand, where AP endonuclease nicks the edited strand and leads to DSBs, bringing indels or cell death. (**B**) ABE consists of impaired Cas9, gRNA, and ecTadA–TadA* homodimer (ecTadA: wild-type tRNA adenosine deaminase from *E. coli*, TadA*: evolved ecTadA that can operate on DNA). ABE deaminates A to I at the target, where I is misread as G by DNA polymerase. (**C**) PE consists of nCas9, reverse transcriptase (RT), and prime editing gRNA (pegRNA). PegRNA comprises two essential parts: PBS (primer binding site) and RTT (reverse transcriptase template). PE nicks the edited strand. The strand then hybridizes with PBS and extends with the copy of RTT by RT so that the mutation is introduced to the strand. The 5′ flap intermediate is removed by flap endonuclease, and the 3′ flap is ligated. The desired edits are consequently introduced into the DNA sequence. (**D**) GBE consists of nCas9, PmCDA1, and UNG in *E. coli*. GBE deaminates the C at the edited strand and nicks the other strand. U is removed by UNG, so the target becomes an AP site where TLS polymerases assemble and extend the nicked strand by Polζ. When passing by the locus opposite to the AP site, Rev1 cooperates with C and Polδ with T, which results in the creation of G:C and A:T, respectively, after DNA repair or replication.

The basic constructs and working mechanisms of CBE and ABE are illustrated in Figure 3A,B. CBE in Figure 3A is named Target-AID, fusing Petromyzon marinus CDA1 (PmCDA1) to the C terminus of d/nCas9 (d/nCas9-PmCDA1). The editing window of Target-AID is commonly at the positions −16 to −20 (counting the PAM as positions 1–3), highlighted in dark red in Figure 3. It is important to note that another type of CBE, not shown in Figure 3, fuses rat APOBEC1 (rAPOBEC1) to the N terminus of d/nCas9 (rAPOBEC1-d/nCas9) with the editing window at −13 to −17. In past years, various versions of CBE and ABE have been developed to overcome the editing limitations, such as low editing efficiency, off-target effects, and PAM requirements, by utilizing Cas9 homologs and variants, deaminase homologs and variants, modifying gRNA, and so on. Their characteristics and applications in animals, plants, and bacteria are concluded comprehensively by excellent reviews [27,30,32,50,51,52]. Anzalone and colleagues also introduced a decision tree for choosing different BEs on the basis of several criteria [38]. Recently, several developed BEs have been reported to further address editing limitations in microbes. For example, PmCDA1 was engineered with intensive truncations, and several mutations were inlaid in the middle of nCas9 [53], which not only performed comparable editing efficiency to Target-AID, but also greatly minimized the off-target effect and molecular size in *S. cerevisiae*. The gRNA structure was engineered with a bubble hairpin that contained a 5′ extended sequence that was complementary to the guide sequence in order to decrease the off-targets of both CBE and ABE in *E. coli* [54]. In *Bacillus subtilis* (*B. subtilis*), CBE has been developed from a primary version dCas9-PmCDA1 with a narrow editing window (−17 to −18) [55] compared with dCas9-PmCDA1-UGI [56], with almost 100% editing efficiency at positions −16 to −20 by adding UGI and replacing the strong promoter P*grac* with a weak one, P*spac*. Furthermore, to overcome the low transformation of nCas9-PmCDA1 [36,55,56], which is probably caused by the toxicity of the fusion protein, the PmCDA1-nCas9-UGI was integrated into the genome and performed with approximately 100% efficiency at positions −15 to −20 [57]. The 20 nucleotides (nt) gRNA was also engineered using extensions or adding an artificial stem loop to increase the editing efficiency and to expand the editable window [57]. The development of CBE and ABE to overcome their limitations in microbes are all concluded in Table 1. 

C:G and T:A are currently interchangeable through the combination of CBE and ABE, which is able to generate 62 different amino acid substitutions [44]. ABE has been used to disrupt genes by converting start codon ATG to ACG in *Streptomyces* [45], which is a good supplement for achieving genome-wide targeting with CBE. However, scientists are still looking forward to achieving DNA base transversion (conversion between purine and pyridine) so that any base can be arbitrarily converted to any other. As newly developed gene engineering technologies, prime editors (PE) and glycosylase base editors (GBE) meet the abovementioned need, and supplement the current use of BEs.

#### 2.1.2. Latest Development of PEs in Microbes

The basic construct and working mechanism of a PE are shown in Figure 3C, as reviews [27,31] have also discussed, but there have been few reports of a PE applied in microbes. Recently, PEs have been established as a versatile gene editing tool in *E. coli* [58]. By optimizing the length of PBS (13~17 nt) and RTT (13 nt), a PE could substitute, insert, and delete chromosome DNA with 6.8%, 12.2%, and 26% efficiency, respectively. However, the introduction of a second gRNA to nick the opposite strand, which further increases the efficiency in mammalian cells [59], led to low transformation, and therefore, compromised the use of PEs in microbes. The dual-editing events also showed low efficiency (<1%). Although PEs achieve highly versatile editing with few byproducts and off-target events, its low efficiency still hinders its broad application in microbes.

#### 2.1.3. Latest Development of GBE in Microbes

nCas9-CBE has been reported to obtain quite a few C to non-T products in *S. cerevisiae* [33], *C. glutamicum* [39], *R. sphaeroides* [48], *Klebsiella pneumoniae* (*K. pneumoniae*) [60], and so on. Inspired by the observations above, Kurt’s group [61] and Zhao’s group [62] fused UNG to the C terminus of BE4max (an improved version of CBE with two UGIs and biparticle SV40 NLS) and rAPOBEC1-nCas9, respectively, to amplify the effect on product impurities. The results both showed a great preference for C-to-G conversion in mammalian cells. Kurt and colleagues further optimized the GBE system with the APOBEC1 variant (R33A) to reach 41.7–71.5% editing efficiency [61]. In the meantime, Zhao and colleagues reported that fusing UNG to the N terminus of nCas9-AID (UNG-nCas9-AID), shown in Figure 3D, preferred to convert C to A in *E. coli* with an average editing specificity of 93.8% ± 4.8%, and an editing efficiency of 87.2% ± 6.9% [62]. 

The different editing preference of GBE in mammalian cells and *E. coli* attracted Jiang and colleagues’ attention. They further revealed this mechanism in *S. cerevisiae* [63], as illustrated in Figure 3D. The essential steps for GBE editing are the AP site formation at the edited strand and the nick at the other strand. Then, the replicative DNA polymerases are recruited to repair the nick, known as translesion synthesis (TLS), which are conserved from bacteria to mammals [64]. By knocking out genes encoding TLS polymerases Pol*ζ*, Rev1, Pol*η*, and Pol*δ*, respectively, Pol*ζ* was proven to work as an extender, Rev1 was shown to specifically insert C at the opposite locus of the AP site, and Pol*δ* was shown to mainly incorporate T or A, whereas Pol*η* was not involved in this process [63]. The different TLS polymerases working on the AP site led to various base conversions, possibly providing new methods to fuse together polymerases with BEs to accomplish any desired conversions in the future. Recently several base excision repair (BER) proteins including DNA polymerase β, DNA ligase III, and XRCC1, were fused separately with rAPOBEC1-nCas9 to manipulate the BER pathway downstream of AP creation, giving rise to G as the major product [65]. Koblan and colleagues investigated the impact of DNA repair proteins on GBE efficiency by using CIRISPR interference (CRISPRi) screens. They engineered new GBEs with diverse editing profiles by fusing various DNA repair proteins, deaminases, and Cas proteins together [66]. Trained in a library of results at 10,638 genomically integrated target sites, the machine learning models, CGBE-Hive, could predict GBE editing efficiency and purity, as well as bystanders’ editing patters with high accuracy. To overcome the limitations of sequence preference and PAM requirement, Chen and colleagues tested a series of new GBEs and argued that the rational design of deaminase, rather than the addition of BER proteins, improved the editing efficiency [67]. The optimized eAID variant-nCas9 fusion protein improved the target compatibility of the GBE system in the GC context [67]. The spCas9 was further replaced with its variants, SpG Cas9 and SpRY Cas9, to expand the targeting range.

**Table 1:** Developed Versions of BEs to Overcome Editing Limitations in Microbes.

BE type	Year	Fusing Enzyme	Cas9 Protein	gRNA	Construct	Improved Editing Activity	Applications	Refs
To narrow the editing window								
CBE	2019	PmCDA1 with a series of C-terminal truncations	nCas9^D10A^	20 nt	PmCDA1 variants-nCas9-UGI	Prefer to edit at positions −17 to −18 while retaining editing efficiency	Edit polyC motifs and Can1 to test narrow editing windows of C-terminal truncations such as CDA1Δ190, Δ192, Δ194 in *S. cerevisiae*	[34]
CBE	2020	APOBEC3A with a series of C-terminal truncations and mutations	nCas9^D10A^	20 nt	APOBEC3A variants-nCas9-UGI	Prefer to edit at positions −15 to −16 with decreased off-target RNA editing	Edit Can1 to test editing activity of APOBEC3A truncations such as Δ182, Δ186 and Δ190 in *S. cerevisiae*	[35]
To expand the editing range								
CBE	2019	PmCDA1	nVQR^D10A^, nVRER^D10A^, nxCas9^D10A^, nCas9-NG^D10A^	20 nt	nCas9 variants-PmCDA1	Recognize the targets at non-NGG PAM with high editing efficiency	Introduce an amino acid transition T311I of LysC to obtain the mutant strain with 1.7 g/L lysine production in *C. glutamicum*	[40]
			nCas9^D10A^	18–30 nt	nCas9-PmCDA1	Increase editing efficiency at positions −14 and −15 by using 18 nt gRNA; increase efficiency at position −21 by using 22 and 24 nt gRNA	Edit poly C motifs in the plasmids and chromosomes of *C. glutamicum* to test the editing window shift by using truncated or extended gRNAs	
CBE	2020	PmCDA1	nVQR^D10A^, nVRER^D10A^, nxCas9^D10A^, nCas9-NG^D10A^	20 nt	PmCDA1 variants-nCas9 variants-UGI	Recognize the targets at non-NGG PAM with a relatively narrow editing window from −17 to −18	Edit polyC motifs to test the availability of Cas9 variants, editing efficiency, and window in *S. cerevisiae*	[35]
CBE	2021	PmCDA1	dCas9	20 nt	dCas9-PmCDA1-UGI	Broaden the editing window from −16 to −20 with 100% efficiency and increase the multiplex gene editing efficiency to 75.5% for quintuple targets by adding UGI	Test five different constructs of CBE in *B.subtilis* by inactivating GFP and multiple genes	[56]
CBE	2021	PmCDA1	nCas9^D10A^	20 nt	PmCDA1-nCas9-UGI (integrated into genome)	Broaden the editing window at position −15 to −20 with 97–100% efficiency	In situ mutate Sec-translocase and BceB protein to obtain mutant strains with 3.6-fold transportation efficiency and different sensitivity to bacitracin, respectively, in *B. subtilis*	[57]
				21–26 nt		Increase editing efficiency by using 21 and 22 nt gRNA; expand editing window from −15 to −22 by using 23–26 nt gRNA;		
				20 nt with a stem loop at 3‘ end of gRNA		increase the editing efficiency at position −15 from 70% to 87%		
To decrease the off-target effect								
CBE	2020	rAPOBEC1 variants	dCas9	20 nt	rAPOBEC1 variants-dCas9-UGI	Balance efficient, on-target editing with greatly decreased gRNA-independent editing	Develop multiple rapid, cost-effective methods to screen the propensity of different deaminase variants and engineer the YE1 variants that retain high editing activity with minimal gRNA-independent off-target editing	[68]
CBE	2021	tCDA1EQ (PmCDA1Δ30-150, W122E, W133Q) tCDA1EQ	nCas9^D10A^	20 nt	tCDA1EQ-nCas9	Significant decrease (5–79 fold) gRNA-independent off-target effects with comparable editing efficiency to original CBE	Edit *Can1* to test editing activity in *S. cerevisiae*, evaluate the editing efficiency and window in mammalian cells, and compare them with existing improved CBEs	[53]
					nCas9 1054aa-tCDA1EQ-1055aa (inlaid architecture)			
CBE	2021	rAPOBEC1	nCas9^D10A^	20nt with H12-B3-P5 (a 3 nt bubble positioned from positions 5 to 7 into a 12 nt hairpin)	rAPOBEC1-nCas9-UGI	Significantly decrease off-target editing without sacrificing on-target editing efficiency	Test editing efficiency and gRNA-dependent off-targets in *E. coli*	[54]
ABE		ecTadA-TadA*		20 nt with H12-B3-P4	ecTadA-TadA*-nCas9			
To achieve DNA base transversion								
PE	2021	M-MLV (reverse transcriptase)	nCas9^H840A^	20 nt with 13–17 nt PBS and 13 nt RTT	nCas9-M-MLV2	Substitutions, insertions, and deletions with 6.8%, 12.2% and 26% efficiency, respectively, in chromosome with few bystanders and off-targets	Achieve gene substitutions, deletions (up to 97 bp), insertions (up to 33 bp), and multiplex editing in *E. coli*	[58]
GBE	2021	PmCDA1	nCas9^D10A^	20 nt	UNG-nCas9-PmCDA1	Convert C to A with an average editing efficiency of 87.2% ± 6.9% with no detectable gRNA-dependent off-target	Convert C to A at four loci in *lacZ* and develop the NBE (any base editing) strategy in *E. coli* by combining CBE, ABE, and GBE	[62]

### 2.2. Recent Applications of BEs in Nonmodel Microbes

BEs have been applied in a wide range of nonmodel microbes. Some BEs perform high editing efficiency, but some of them can barely function in microbes; therefore, we present a detailed list of all constructs and applications of BEs in nonmodel microbes, shown in Table 2. The latest applications that have not been reviewed before are discussed below. It is important to note that the editing activities of BEs described in Table 2 have some differences with the descriptions in previous reviews [30,31]. For example, the efficiency of CBE in *Streptomyces* [44] is described as 30–100% in Table 2, which is based on the original text, “Overall, the cytidines in the editing window were converted into thymidines with frequencies between 30% and 100%” [44], whereas Wang et al. [30] chose the highest efficiency, 100%, in *Streptomyces*. The efficiency of ABE in *Streptomyces* [44] is described as 0–100% in Table 2, based on Figure 2D from the original paper [44], from the lowest efficiency, 0, to the highest efficiency, 100%. Although Jiang et al. [31] chose the result in Supplementary Material Figure S1.B from the original paper [44], which showed the editing efficiency was from 0 to 40% [44]. Similarly, the efficiency in *Clostridium beijerinckii* is 20–100% in Table 2, whereas it is 40–100% in Jiang et al. [31] The lowest efficiency in Table 2 is based on the result of Figure 5a in the original paper [47], which shows only one mutant is successfully edited from the five picked colonies. Jiang et al. chose the result described in original text; “Nine out of those twenty clones obtained from pCBEclos-cbei1006-g2 which grew on 5-FOA medium were all shown by Sanger sequencing of amplified PCR products, to contained the desired mutations”.

**Table 2:** The Characteristics and Applications of BEs in Nonmodel Microbes.

Species	Major Function	Type	Year	gRNA Promoter	Construct of Fusion Protein	PAM	Editing Window	Editing Efficiency	Multiplex Gene Editing	Applications of BEs	Off-Targets	Refs
Industrially Important Microbes												
*Kluyveromyces marxianus*	Industrial production of various enzymes, chemicals, and macromolecules, as well as the utilization of cell biomass	CBE	2017	P_SNR52_	PTSNR52	NGG	−17 to −18	12.5–25%	nr	Inactivate *Nej1* and *Dnl4* to build NHEJ null mutants with an increased efficiency of homologous recombination and to facilitate multiple integration mediated by CRISPR/Cas9	nr	[69]
*Psedomonas* spp.	An excellent bacterial host to produce polymers, bulk chemicals, drugs, and high-price specialties	CBE	2018	P_trc_	P_rpsL_-rAPOBEC1-nCas9^D10A^	NGG	−13 to −18	100%	nr	Inactivate genes in *P. aeruginosa* PAO1, *P. putida* KT2440, *P. fluorescens* GcM5-1A, and *P. syringae* DC3000 to test editing window and efficiency	nd in the six similar spacers of the *rhlR* and *rhlB* genes	[41]
		CBE	2020	P_j23119_	P_bs_/P_araBAD_-rAPOBEC1-nCas9^D10A^	na, none of the selected colonies achieved C-to-T mutations						[70]
		CBE	2020	P_j23119_	P_bs_-rAPOBEC1-eSpCas9pp^D10A^							
		CBE	2020	P_j23119_	P_araBAD_-rAPOBEC1-eSpCas9pp^D10A^	NGG	nr	20%	nr	Edit *ttgA* to test editing efficiency	nr	
		CBE	2020	P_j23119_	P_m_-rAPOBEC1-eSpCas9pp^D10A^-UGI	NGG	−13 to −18	40–60%	nr		nr	
		CBE	2020	P_j23119_	P_araBAD_-rAPOBEC1-eSpCas9pp^D10A^-UGI	NGG	−13 to −18	80–100%	100% for double targets and 35% for triple targets	Inactivate genes in *P. putida*, *P. aeruginosa*, *P. fluorescens*, and *P. entomophila* to prove CBE general availability; simultaneously edit genes by one-plasmid and two-plasmid system	nd by Sanger sequencing the potential sites predicted by CasOT	
		CBE	2020	P_j23119_	P_araBAD_-rAPOBEC1-eSpCas9pp^D10A^-NG-UGI	NG	−13 to −18	100%	100% for double targets recognized by eSpCas9pp and eSpCas9-NG in a two-plasmid system	Inactivate *pykA* and *pcaH* in one step; mutate G136 in AroF-2 to select a mutant strain with increased PCA titer up to 264.87 mg/L	nr	
		CBE	2020	P_j23119_	P_araBAD_-YE1-eSpCas9pp^D10A^-UGI	NGG	−14 to −17	62.5%	nr	Precisely edit *ttgA*, which contains multiple cytidines with enhanced editing efficiency from 25% to 62.5%	nr	
*Yarrowia lipolytica*	GRAS and industrial production of lipase and organic acids	CBE	2019	P_SCR1’-tRNA_^Gly^	P_UAS1B8-TEF(136)_-nCas9^D10A^-PmCDA1-UGI	NGG	−14 to −20	28%	6.7% for double targets	Inactivate *TRP1*, *PEX10*, *HIS3* in *ku70*Δ strain to test editing efficiency	nr	[26]
		CBE	2019	P_SCR1’-tRNA_^Gly^	P_TEFin_-nCas9^D10A^-PmCDA1-UGI	NGG	−14 to −20	94.3 ± 5%	31% for double targets		nr	
*Streptomyces* spp.	Industrial production of bioactive secondary metabolites, such as antifungals, antivirals, antitumorals, anti-hypertensives, and mainly antibiotics, etc.	CBE	2019	P_ermE*_	P_tipA_-rAPOBEC1-nCas9^D10A^-UGI	NGG	−11 to −17	30–100%	33.3% for triple targets	Substitute amino acids in SCO5087 and SCO5092; inactivate genes of BGCs in nonmodel strain *S. griseofuscus*; efficiently and simultaneously inactivate two identical copies of *kirN*	38–56 by WGS (24–34 meaningful amino acid changes); whereas 29 SNVs in wild-type strain (18 amino acid changes);	[44]
		ABE	2019	P_ermE*_	P_tipA_-TadA-TadA*-nCas9^D10A^-UGI	NGG	−12 to −17	0–100%	nr	Target SCO5087 and a designed matrix containing NA motifs to test efficiency and preference	27–33 by WGS (20–21 meaningful amino acid changes)	
		CBE	2019	P_kasO*_	P_rpsL_-rAPOBEC1-dCas9-UGI	NGG	−13 to −17	43–70%	43% for double targets	Edit *redD* and *actl* to test C-to-T efficiency with a few C-to-G and C-to-A mutations	nr	[45]
		CBE	2019	P_kasO*_	P_rpsL_-rAPOBEC1-nCas9^D10A^-UGI	NGG	−13 to −17	100%	100% for double targets; 60% for quintuple targets	Simultaneously disrupt the genes of polyketide synthase clusters to increase production of avermectin	3 by Sanger sequencing the sites predicted by CasOT;	
		CBE	2019	P_kasO*_	P_rpsL_-rAPOBEC1-HF-nCas9^D10A^-UGI	NGG	−13 to −17	80%	nr	Edit *olm* to test off-target events, which was decreased to an undetectable level	nd by Sanger sequencing the sites mentioned above	
		ABE	2019	P_kasO*_	P_rpsL_-TadA-TadA*-dCas9	na, all selected colonies showed the A/G overlapping peak in sanger sequencing						
		ABE	2019	P_kasO*_	P_rpsL_-TadA-TadA*-nCas9^D10A^	NGG	−14 to −17	100%	nr	Disrupt the initiation of *actVB* translation by converting ATG start codon to ACG to accumulate actinoperylone	nr	
		CBE	2019	P_j23119_	P_ermEp*_-dCas9-PmCDA1-UL	na, growth of cells is severely delayed when CBE was overexpressed by the strong constitutive promoter						[46]
		CBE	2019	P_j23119_	P_tipAp_-dCas9-PmCDA1-UL	NGG	−16 to 20	10–100%	60% for double targets; 20% for triple targets	Inactivate genes in *S. coelicolor* and *S.rapamycinicus* to test editing efficiency and general availability to other strains	1 by Sanger sequencing the potential sites predicted by Cas-OFFinder	
		CBE	2019	P_j23119_	P_tipAp_-nCas9^D10A^-PmCDA1-UL	NGG	−16 to 20	15%	nr	Edit *redW* with low efficiency from C to T but 85% efficiency for C-to-G mutation	nr	
		CBE	2021	P_gapdh (EL)_	P_rpsL(XC)_-rAPOBEC1-dCas9-UGI	NGG	−13 to −18	1–20%	nr	Edit *redN*, *redD*, and *act_β-ketoacyl* to test editing efficiency	16.50 ± 8.35 by WGS	[71]
		CBE	2021	P_gapdh (EL)_	P_rpsL(XC)_-rAPOBEC1-nCas9^D10A^-UGI	NGG	−13 to −18	3–25%	nr		nr	
		CBE	2021	P_gapdh (EL)_	P_rpsL(XC)_-rAPOBEC1-dCas9-UGI with asRNA	NGG	−13 to −18	21.2–65.8%	nr		13.50 ± 3.32 by WGS	
		CBE	2021	P_gapdh (EL)_	P_rpsL(XC)_-rAPOBEC1-nCas9^D10A^-UGI with asRNA	NGG	−13 to −18	26.2–79.4%	nr		nr	
*Clostridium beijerinckii*	Production of acetone, n-butanol, isopropanol etc.	CBE	2019	P_j23119_	P_thl_-rAPOBEC1-nCas9^D10A^-UGI	NGG	−13 to −17	20–100%	nr	Edit *pyrE*, *xylR*, *spo0A*, and *araR* to test efficiency of codon-optimized CBE; inactivate *xylR* to enhance the xylose fermentation	nr	[47]
*Clostridium ljungdahlii*	Production of acetic acid and ethanol from waste gas	CBE	2020	P_j23119_	P_2TetO1_-dCas9-PmCDA1-UL	NGG	−11 to −19	2–55.6%	nr	Inactivate *adhE1* and *aor2* separately to increase acetate yield as well as lower ethanol production under either heterotrophic or autotrophic conditions	nr	[72]
*Rhodobacter sphaeroides*	Industrial production of CoQ10, isoprenoids, poly-β-hydroxybutyrate, hydrogen	CBE	2020	P_j23119_	P_Lac_-dCas9-PmCDA1-UL	NGG	−14 to 20	16.7%	nr	Inactivate *appA* and *ppsR* to test efficiency with pure C-to-T conversion	nr	[48]
		CBE	2020	P_j23119_	P_Lac_-nCas9^D10A^-PmCDA1-UL	NGG	14 to 20	10–96.7%	43% for double targets; 46.7% for triple targets	Inactivate *appA*, etc., to test C-to-T efficiency with C-to-G and C-to-A byproducts; disrupt *ubiF*, *ubiA*, *ubiG*, and *ubiX* to reveal their importance in the CoQ10 biosynthetic pathway	nr	
		ABE	2020	P_j23119_	P_Lac_-TadA-TadA*-dCas9	NGG	−14 to −16	0–5%	nr	Edit *appA*, *ppsR*, *crtB*, and *bchG* to alter translation level or block translation initiation	nr	
		ABE	2020	P_j23119_	P_Lac_-TadA-TadA*-nCas9^D10A^	NGG	−14 to −16	0–30%	nr	Edit *appA*, etc to alter translation level or block translation initiation	nr	
*Shewanella oneidensis*	Bioelectricity production from biomass wastes	CBE	2020	P_tac_	P_rpsL_-rAPOBEC1-nCas9^D10A^	NGG	−13 to −18	33.3–100%	87.5% for double targets	Target NC motifs to test editing preference; inactivate *gfp*, *blaA*, and *dmsE* to test editing activity; identify key genes in GlcNAc or glucose metabolism to obtain a mutant strain with enhanced degradation efficiencies for organic pollutants	nr	[49]
*Companilactobacillus crustorum*	Production of bacteriocin and 3-phenyllactic acid	CBE	2021	P_3_	P_sppA_-rAPOBEC1-nCas9^D10A^	NGG	−14 to −18	75–100%	nr	Edit seven C-rich spacer sequences in a plasmid to test editing window and efficiency	nr	[73]
**Agriculturally Important Microbes**												
*Paenibacillus polymyxa*	Nitrogen fixation, plant growth promotion, soil phosphorus solubilization and production of cxopolysaccharides, hydrolytic enzymes, antibiotics, and cytokinin	CBE	2021	P_ara_	P_grac_-nCas9^D10A^-PmCDA1	na, no transformant was obtained due to the toxicity of the fusion protein						[56]
		CBE	2021	P_ara_	P_spac_-dCas9-PmCDA1-UGI	NGG	−16 to 20	100%	100% for double and triple targets; 83.3% for quadruple targets; 75.5% for quintuple targets	Disrupt genes of five known BGCs to reveal the antimicrobial spectrum of the novel antibiotics in the sixth unknown BGCs	8.5 SNVs including 4.2 amino acid changes by WGS	
*Agrobacterium* spp.	Nature’s genetic engineer for diverse species including crops	CBE	2021	P_j23119_	P_aadA_-dCas9-PmCDA1-UGI-LVA	na, no correct clones were obtained in *E. coli* probably due to the toxicity						[74]
		CBE	2021	P_j23119_	P_virB_-dCas9-PmCDA1-UL	NGG	−15 to −19	91%	80% for double targets	Inactivate *recA* to maintain stability for plant transformation; separately inactivate *rolB*, *rolC*, and *orf13* to confirm their importance in hair root construction	nr	
*Sinorhizobium meliloti*	Perform symbiotic nitrogen fixation within leguminous host plants such as alfalfa, an important forage crop	ABE	2021	P_SigA/_P_RpoN_/P_tyr_	P_HemA_-TadA-TadA*-nCas9^D10A^	na, failed to mediate the A-to-G transition when gRNA is expressed by promoter *SigA*, *RpoN* or *tyr*						[75]
		ABE	2021	P_RpsT_	P_HemA_-TadA-TadA*-nCas9^D10A^	NGG	−11 to −17	60%	nr	Edit *nodA* to test the editing efficiency	nr	
		ABE	2021	P_RpmJ_	P_HemA_-TadA-TadA*-nCas9^D10A^	NGG	−11 to −17	100%	90% for triple targets	Edit *nodA*, *nodB*, *nodC, nifD*, *nifH,* and *nifK* to test if the promoters can drive the expression of the fusion protein to perform efficient editing	nd by Sanger sequencing the potential sites predicted by Cas-OFFinder	
		ABE	2021	P_RpmJ_	P_Neo_-TadA-TadA*-nCas9^D10A^	NGG	−11 to −17	100%				
		ABE	2021	P_RpmJ_	P_Tau_-TadA-TadA*-nCas9^D10A^	NGG	−11 to −17	80%				
		CBE	2021	P_RpmJ_	P_HemA_-rAPOBEC1-nCas9^D10A^-UGI	NGG	−13 to −17	75%	nr	Inactivate *nodA* (W7*) to test if the growth of plants inoculated with the mutant strain was retarded		
		CBE	2021	P_RpmJ_	P_Tau_-rAPOBEC1-nCas9^D10A^-UGI	NGG	−13 to −17	100%	nr			
		CBE	2021	P_RpmJ_	P_HemA_-nCas9^D10A^-PmCDA1-UGI	NGG	−13 to −20	100%	80% for double targets; 50–70% for triple targets	Edit *nodA*, etc to test editing efficiency		
		GBE	2021	P_RpmJ_	P_HemA_-nCas9^D10A^-PmCDA1-UNG	NGG	−14 to −18	33–80%	nr		nr	
**Clinically Important Microbes**												
*Brucella melitensis*	The most important agent of human brucellosis	CBE	2018	P_LlacO-1_	P_trc_-rAPOBEC1-nCas9^D10A^-UGI-NLS	NGG	−15	100%	nr	Inactivate *virB10* by targeting three sites with 100% editing efficiency at only one site	nr	[37]
*Klebsiella pneumoniae*	Cause pneumonia, bloodstream infections, wound, or surgical site infections and meningitis; biosynthesize 1,3-propanediol and 2,3-butanediol	CBE	2018	P_j23119_	P_rpsL_-rAPOBEC1-nCas9^D10A^	NGG	−13 to −18	100%	nr	Edit *fosA* and *dhaK* to test editing efficiency with a few C-to-A byproducts; inactivate the *bla*_KPC-2_ and *bla*_CTX-M-65_ to dissect drug-resistance mechanisms	nr	[60]
*Staphylococcus aureus*	Cause infections ranging from skin infections to severe systemic infections	CBE	2018	P_cap 1A_	P_rpsL_-rAPOBEC1-nCas9^D10A^	NGG	−13 to −17	100%	nr	Inactivate *agrA* and *cntA* to test efficiency	nr	[76]
		ABE	2020	P_cap 1A_	P_rpsL_-ecTadA-TadA*-nCas9^D10A^	NGG	−13 to −17	50–100%	100% for double targets	Screen key residues of cntBC targeted by 38 gRNAs to obtain 42 mutant strains	nd gRNA-dependent off-target by WGS	[77]
*Acinetobacter baumannii*	causing ventilator-associated pneumonia and bloodstream infections, and mortality rates can reach 35%	CBE	2019	P_j23119_	P*tac*-rAPOBEC1-nCas9^D10A^	NGG	−13 to −18	20–100%	nr	Edit *tynA*, *acel*, *adeB*, *cpdA*, *entE*, and *oxyR* to test editing efficiency and preference of TC motifs; disrupt drug-resistance relevant genes *blaOXA-23*, *blaTEM-1D*, and *blaADC-25* to dissect drug-resistance mechanisms	nr	[78]
*Mycobacterium* spp.	Causes tuberculosis, getting 10 million infections and 1.45 million deaths in 2018 worldwide	CBE	2021	P_j23119_	P_tetR_-rAPOBEC1-dSt1Cas9	NNRGAA	nr	4–15%	nr	Test availability of dSt1Cas9-BE in *Mycobacterium* with low efficiency for C-to-T but 18–70% efficiency for C-to-G	nr	[79]
		CBE	2021	P_j23119_	P_tetR_-rAPOBEC1-dSt1Cas9-UGI-UGI	NNRGAA	nr	12–95%	nr	Inactivate *katG* to obtain mutant strain with increasing resistance to Isoniazid treatment	nr	
		CBE	2021	P_j23119_	P_tetR_-rAPOBEC1-dSt1Cas9_evolve_-UGI-UGI	NNNNAA	−10 to −14	20–95%	87.5% for both double and triple targets	Inactivate the essential L-leucine biosynthesis genes *leuB* and l*ueC;* inactivate *ctpE* to increase bacterium aggregation in the presence of EGTA	nd gRNA-dependent off-target by WGS	
		GBE	2021	P_j23119_	P_tetR_-rAPOBEC1-dSt1Cas9-UNG	NNRGAA	nr	100%	nr	Edit five different loci to test editing efficiency	nr	
		GBE	2021	P_j23119_	P_tetR_-rAPOBEC1-dSt1Cas9_evolve_-UNG	NNNNAA	−13 to −16	20–65%	75% for triple targets	Edit 29 endogenous genomics sites to find only TC motif is available for editing	nr	
		CBE	2021	P_j23119_	P_tetO_-rAPOBEC1-dSt1Cas9-UGI	NNAGGAC	nr	1.2%	nr	Inactivate *gfp* to test editing efficiency	nr	[80]
		CBE	2021	P_j23119_	P_tetO_-rAPOBEC1-nSt1Cas9-UGI	NNAGGAC	nr	10.3%	nr		nr	
		CBE	2021	P_j23119_	P_tetO_-rAPOBEC1-nSt1Cas9-UGI with assistant plasmid expressing recX	NNAGGAC	−12 to −18	37.5–100%	nr		nr	
		CBE	2021	P_j23119_	P_tetO_-rAPOBEC1-nSt1Cas9-UGI with assistant plasmid expressing recX and nucS_E107A_	NNAGGAC	nr	12.5–75%	nr	Inavtivate *Rv0582*, *Rv0627* and *Rv2530* to test efficiency; Inactivate *katG* to build a mutant stain with higher 50% minimum inhibitory concentration than the wild-type strain	nr	

nr: not reported, nd: not detected, na: not available, UL: UGI-LVA (protein degradation tag), SNVs: single-nucleotide variants, WGS: whole-genome sequencing, GRAS: generally recognized as safe, BGC: biosynthetic gene cluster. **The construct of BEs that failed to work in microbes are marked in red.**

#### 2.2.1. Industrially Important Nonmodel Microbes

##### *Kluyveromyces marxianus* 

As a nonmodel yeast with high growth rate, thermotolerance and a wide sugar assimilation spectrum, *Kluyveromyces marxianus* (*K. marxianus*) has great potential for industrial applications to produce various enzymes, chemicals, ethanol, and so on [81]. The high activity of the NHEJ system in *K. marxianus* hinders the development of CRISPR/Cas9; therefore, an alternative and efficient gene editing method is required to accelerate its genetic and metabolic characterizations. After Target-AID was established in *S. cerevisiae* [33], it was further applied in *K. marxianus*, belonging to the same class with *S. cerevisiae*, to inactivate NHEJ-related genes, *Nej1* and *Dnl4* [69]. The NHEJ null mutants with enhanced HR events facilitated the markerless integration mediated by CRISPR/Cas9. Although the mutants were successfully obtained, the editing efficiency of CBE was quite low, which can be improved in the future. With the combination of BEs and CRISPR/Cas9, this nonmodel species can be further explored for industrial applications.

##### *Psedomonas putida* 

*Psedomonas* is a widespread genus from all over the world, especially found in extreme environments. It is reported to have strong potential for detoxifying environmental pollutants [82] and producing industrial bioactive compounds and pharmaceutical proteins. *Psedomonas putida* (*P. putida*) was used to produce monoterpene folic acid, fatty alcohols, rhamnolipids (Rs), and polyhydroxyalkanoates (PHAs) [83]. *P. aeruginosa* and *P. fluorescens* were reported to be the best-characterized phenazine producers as well [83]. After CBE, rAPOBEC1-nCas9, was proven to be able to inactivate genes in the *Psedomonas* species [43], and researchers were committed to improving its editing activity. Sun and colleagues [70] established a developed CBE, fusing rAPOBEC1 and UGI with an enhanced specificity nCas9 variant (eSpCas9pp^D10A^, containing mutations K848A/K1003A/R1060A) to fulfill almost 100% editing efficiency with no detectable gRNA-dependent off-target event in *P. putida* KT2440, *P. aeruginosa* PAO1, *P. fluorescens* Pf-5, and *P. entomophila L48*. Furthermore, similar to previous studies [35,40], using Cas9 variants to extend editable sites in the genome, eSpCas9pp^D10A^ was engineered with additional mutations (L1111R, D1135V, G1218R, E1219F, A1322R, R1335A, T1337R), named eSpCas9pp^D10A^-NG, recognizing the NG PAM site in *P. putida* with 100% editing efficiency. rAPOBEC1 was also replaced with YE1[68] to narrow the editing window to nearly 2 nt in case unwanted conversions were created at other cytidine sites and led to low editing efficiency at the target [70]. Variants of deaminase enzymes can also be applied to other microbes to achieve precise conversion when the editing window comprises multiple cytidines.

##### *Streptomyces lividans 66* 

*Streptomyces,* as a well-known antibiotic producer, plays an important role in producing more than two-thirds of medically and agriculturally important secondary metabolites, such as polyketides and nonribosomal peptides [84]. However, the production of many microbial drugs is very low in original strains since most BGCs are not or poorly expressed under traditional laboratory conditions. The solutions can be knocking out the competitive pathways, replacing negative promoters with strong ones, overexpressing positive regulatory genes [85], etc., which are all manipulated by efficient gene engineering techniques. Primary works have made great developments with CBE and ABE in *Streptomyces* to achieve single gene inactivation, multiplex gene editing, and reduction of off-target effects by fusing the high fidelity Cas9 variant [44,45,46]. Recently, Zhang and colleagues [71] developed an antisense RNA (asRNA) interference-enhanced CBE to increase editing efficiency in *Streptomyces lividans* 66 (*S. lividans* 66) since rAPOBEC1-d/nCas9-UGI in *S. lividans* 66 demonstrated a much lower editing efficiency than that in *Streptomyces colicolor*, shown in Table 2. They verified that the deletion of *ung*1 in *S. lividans* could significantly improve the editing efficiency without toxicity. Considering that the permanent inactivation of *ung* might be detrimental for industrial applications, they added an asRNA with CBE to transiently downregulate UNG which could become a normal level after plasmid curing. This design showed an efficiency enhancement of approximately 2.8 to 65.8 times compared with the original CBE and was more controllable than gene deletion. This construct can additionally be applied in other species, especially for those with a low editing efficiency of CBE.

##### *Clostridium ljungdahlii* 

*Clostridium*, as a predominant cluster of commensal bacteria in the human gut, has an important place in biofuel and chemical production thanks to its unique capability of utilizing virtually all biomass-derived carbohydrates and waste gases [86]. Although the CRISPR/Cas9 technique has made great advancements in producing precise and fast gene editing in these species [87], limitations still exist such as the low transformation of bulky plasmids comprising large donor templates and low HR efficiency in some species [88]. Previously, Li and colleagues first established a CBE system, rAPOBEC1-nCas9-UGI, in *C.beijerinckii* [47]. The success of CBE working in *C. beijerincki* brought hope to its application in other species, such as *C. ljungdahlii*, which is popular for converting inorganic one-carbon (C1) gases into industrially important products, such as acetate and butanol [89], but is reported to be very inefficient and not robust for foreign DNA transformation [90].

In 2020, the carbon flux of ethanol production in *C. ljungdahlii* was reprogrammed by CBE to improve acetate production [72]. Researchers applied a relatively mild construct dCas9-PmCDA1-UL, rather than rAPOBEC1-nCas9-UGI, to lower the expression of CBE in *C. ljungdahlii*. By using a relatively loose linker (121 amino acids), CBE was able to edit within positions −2 to −19 of the protospacer and it exhibited the highest efficiency between positions −16 to −19 in *C. ljungdahlii*. Position −20 was not observed as being edited despite 20 nt and 22 nt gRNA being used, which could be investigated later with more targets or longer gRNAs. The genes involved in ethanol production were inactivated step by step to engineer strains with higher acetate yields, which could be accelerated by multiplex gene editing in the future. Although *C. ljungdahlii* is an A-T rich bacterium, it still comprises 99.83% editable sites for CBE to introduce missense mutations, nonsense mutations, or premature stop codons, estimated by a genome-scale algorithm [72]

##### *Companilactobacillus crustorum* 

*Companilactobacillus crustorum* (*C. crustorum*, formerly named *Lactobacillus crustorum*), a newly isolated lactic acid bacterium from koumiss, is regarded as a novel probiotic agent because multiple components of the antimicrobial peptide transport system were discovered in it [91], such as 3-phenyllactic acid (PLA), which is a broad-spectrum antimicrobial compound that is widely used in the food and textile industries [92]. Unfortunately, *C. crustorum* has been poorly studied due to the lack of an efficient genetic method. Wang and colleagues recently established the CRISPR/Cas9 and CBE system in *C. crustorum* to identify the role of 12 bacteriocin-encoding genes [73]. rAPOBEC1-nCas9 was available to edit the C-rich spacer sequences in plasmid with almost 100% editing efficiency at positions −14 to −18. Multiplex gene editing using BEs can be further applied in chromosomes to investigate probiotic gene clusters and to characterize the unknown metabolic pathways in *C. crustorum* and others in the *Lactobacillus* family.

#### 2.2.2. Agriculturally Important Nonmodel Microbes

##### *Paenibacillus polymyxa* 

*Paenibacillus polymyxa* (*P. polymyxa*, formerly known as *Bacillus polymyxa*) is an agriculturally important microbe as it can directly promote crop growth via nitrogen fixation and phosphate solubilization [93]. It naturally produces a large number of valuable compounds like exopolysaccharides (EPS), 2,3-butanediol, antibiotics, and antimicrobial peptides such as polymyxin and fusaricidin [94]. Since the traditional gene editing methods had a low efficiency and required the integration of a selection marker, the discoveries of the unknown characteristics and physiochemical properties of *P. polymyxa* need a robust and efficient gene editing method.

Recently, after editing activities of different CBEs were compared with *B. subtilis* [56], dCas9-PmCDA1-UGI, which has the best editing efficiency, was also applied in *P. polymyxa*. There are six BGCs in the *P. polymyxa* genome and five of them are known to produce antibiotics [56]. After the five known BGCs were inactivated by CBE multiplex gene editing, the uncharacterized polyketide produced by the sixth unknown BGC was evaluated. The establishment of CBE in *B. subtilis* and *P. polymyxa* would greatly accelerate the discovery of hidden antibiotics and could be further applied in other *Baccilus* and *Paenibacillus* species. To the best of our knowledge, fusing Cas9 variants with BEs to recognize more PAM sites has not yet been reported in *Baccilus* and *Paenibacillus* species, which can be further explored in order to greatly reduce the number of noneditable genes.

##### *Agrobacterium* spp.

*Agrobacterium*, as a remarkable soil phytopathogen, can achieve a stable transformation using any gene of interest in the plant genome via delivery of transferred (T)-DNA [95]. With the help of a *Agrobacterium*-mediated transfer, CRISPR/Cas9 and BEs have been successfully established in plants to modify genomes for both biological functional analysis and crop improvement [96,97]. However, there has been a lack of characterization of genetic parts in *Agrobacterium* and poor exploration of its metabolic and physiological functions [98]. To improve the transformation of *Agrobacterium* in plants, which was previously reported to be inefficient [99,100], understanding and modifying transformation-related genes in *Agrobacterium* are also important. CBE has recently been established in two widely used plant transformation strains, *A. tumefaciens* and *A. rhizogenes* [74].

When CRISPR/Cas9 was found to be lethal in *A. tumefaciens* [74], the researchers set their sights on CBE, aiming to avoid the lethality caused by DSBs. The construct of dCas9-PmCDA1-UL, by modulating the length of gRNA from 20 nt to 18 nt, was proven to be available for gene editing with at least 98% efficiency in *A. tumefaciens* and *A. rhizogenes*. Interestingly, the CBE that was driven by the constitutive promoter was probably lethal to *E. coli* since no correct clones were obtained. After switching to the AS-activated promoter *virB*, the system could be successfully cloned in *E. coli* and worked well in *Agrobacterium* with or without AS treatment. The inactivation of *rolB* and *rolC* was efficiently achieved to confirm their importance in hairy root development. CBE facilitates the understanding and engineering of nature’s engineer, *Agrobacterium*. Further applications of BEs can be used in the field of plant-microbe interactions that help deep learning and improvement of agricultural plants and microbes for beneficial use.

##### *Sinorhizobium meliloti* 

*Sinorhizobium meliloti* (*S. meliloti*) is a soil bacterium that can form nitrogen-fixing nodules on the roots of leguminous plants. The varied and rich metabolic capabilities of *S. meliloti*, such as carbohydrate metabolism, allow it to adapt to very different environmental and nutritional conditions in free-living form or as a plant symbiont [101]. Past studies have reported that *S. meliloti* can be successfully cultivated using starch industry wastewater as feedstock [102], produce cellulase using waste tobacco as a substrate [103], and can be engineered as a high-yield vitamin B_12_ strain [104]. It is also important to engineer its high nitrogen fixing capacity for the benefit of plant survival and reducing environmental toxicity.

Recently, single-plasmid CRISPR-mediated base editing tools (CBE, ABE, and GBE) have all been established in *S. meliloti* [75], which are saving a lot of time and labor compared to traditional tools such as Cre/loxP [105]. After testing a series of promoters for gRNA, the researchers found that an *pRpmJ* promoter (P_RpmJ_) performed a better editing activity than other promoters. ABE, TadA-TadA*-nCas9, was driven by P_HemA_, P_Neo_, or P_Tau_, all of which performed nearly 100% editing efficiency and showed a preference for TA combinations over others, which is consistent with the specificity of ABE in *Streptomyces coelicolor* [44]. CBE and GBE were both driven by P_HemA_ because it could drive the fusion proteins with sufficient efficiency without an inducer. With respect to the CBE used in *S. meliloti*, PmCDA1-CBE exhibited a slightly higher editing efficiency and a wider editing window than rAPOBEC1-CBE, shown in Table 2. After the inactivation of *nodA* in *S. meliloti*, the growth of plants inoculated with the mutant strain was retarded under nitrogen limiting conditions, which proved that *nodA* played an essential role in nitrogen fixation. GBE, nCas9-PmCDA1-UNG was reported to dominantly convert C to A in *E. coli* [62], but instead preferred to produce C-to-G with 30%~80% efficiency in *S. meliloti*. The editing specificity of C-to-G was lower than that of C-to-T. There was no detectable off-target event caused by CBE and ABE, both of which could simultaneously edit multiple genes with 50–90% efficiency. Through whole-genome prediction, there was a 96% editable stop site located in the first 80% of the coding region in *S. meliloti*, showing that BEs have promising potential for high-throughput genome engineering [75].

### 2.3. Clinically Important Nonmodel Microbes

CBE has been established in many human pathogens, including *Brucella melitensis* [37], *P. aeruginosa* [43], *K. pneumoniae* [60], *Staphylococcus aureus* (*S. aureus*) [76], and *Acinetobacter baumannii* [78]. It is worth mentioning the applications in them because the use of BEs can accelerate the understanding of bacterial physiology and drug-resistant mechanisms to develop novel therapeutic strategies.

Recently, ABE was developed as an effective screening tool in *S. aureus* to explore the functional residues of CntBC, a staphylopine/metal complex transporter [77]. ABE 7.10 was linked with 38 different spacers to mutate *cntBC* at various sites separately so that 42 mutant strains were acquired. The growth curves of these strains were tested because the mutations of CntBC residues might relieve the toxicity under a high concentration of cobalt, whereas the overload of cobalt restrained the growth of the wild strain. Through the ABE system, the key residues of CntBC were identified. Multiplex gene editing can be further applied to explore functional genes and new treatments.

*M. tuberculosis* (*Mtb*) is also a therapeutic challenge because of its high resistance to antibiotics [106]. New strategies against *Mtb* infections are urgently needed but are hampered by the unknown genetic backgrounds of the species. The lack of a compatible HR system limits the application of CRISPR/Cas9 in *Mtb*. Fortunately, BEs have recently made great developments in *Mycobacterium* to achieve highly desirable editing. Zhang and colleagues reported that catalytically inactive *Streptococcus thermoplilus* Cas9 (dSt1Cas9) fused with CBE was available to produce C-to-T and C-to-G conversions with 4–15% and 18–70% efficiency, respectively, compared with no detectable base editing by dspCas9- and dLbCpf1- CBE [79]. To overcome the impure editing products, two UGIs and UNGs were separately linked to the C terminus of dStlCas9, yielding dSt1Cas9-CBE, converting C to T with 69–86% efficiency, and dSt1Cas9-GBE, converting C to G with 100% efficiency, respectively. To expand the strict PAM sequence (5′-NNRGAA-3′, R = A/G), wild-type St1Cas9 was replaced with a KQKL variant (St1Cas9_evolve_) containing multiple substitutions with 5′-NNNNAA-3′ PAM specificity, which can inactivate more than 75% open reading frame (ORF) in mycobacterium species by introducing at least one premature stop codon within the top 75% of the ORF body [79]. Later, Ding and colleagues [80] proved that the inhibition of *recA* in HR, and *nucS* in MMR, by expressing RecX and NucS_E107A_ in assistant plasmids, could facilitate CBE efficient editing in *Mtb*. Further manipulation of DNA polymerases to increase the editing activity of BEs could be applied in other species.

## 3. Current Obstacles of BEs in Microbes

### 3.1. Limitation of Editing Activity

As illustrated in Table 2, the multiplex gene editing and off-target effects are still the major problems of BEs. Only a few BEs can simultaneously edit up to five targets in nonmodel microbes [45,56]. Most BEs just achieve double or triple simultaneous editing with low efficiency. One reason might be the technical limitation of creating long arrays of gRNAs. The different efficiencies at various targets are also one of the reasons, because BEs are reported to perform almost 100% editing efficiency at some targets, although they are unable to work at some sites, probably due to the essentiality of genes [44,48,57]. The other serious reason is that as the number of gRNAs increases, they must compete for a dwindling pool of fusion protein, which in turn decreases the efficiency of every gRNA [107]. This problem widely exists in CRISPR technologies and can be possibly solved by using conditional gRNAs, which are selectively triggered as needed in vivo [107].

The off-target effects of BEs also need to be considered and surmounted in the future. The gRNA-dependent off-targets, based on mismatch annealing of gRNAs, can be decreased to an undetectable level by replacing spCas9 with high-fidelity Cas proteins [45], modifications of gRNA structure [54], etc., which are the same strategies used to decrease the off-targets of CRISPR/Cas9 [108]. However, the gRNA-independent off-targets are major and specific problems for BEs. They are thought to be nonspecific and random deamination by the deaminase domain. The gRNA-independent off-targets commonly happen at the transcribed regions because the R-loops formed by the exposure of single-stranded DNA by RNA transcription is a preferred substrate for the deaminases [53]. The on-target activities of BEs still need to be improved in the future. Currently, the structural engineering of deaminases to improve their specificity is one of the solutions, illustrated in Table 1. The expression levels and duration of BEs can also be optimized to mitigate the gRNA-independent off-targets [32]. One-step multiplex editing is recommended, rather than iterative editing, to minimize the off-target effects as the number of targets does not have a large impact on the off-target mutations [56].

### 3.2. Variability of Base Editing Activity

Although BEs generally possess 5 nt editing windows, narrower or broader editing windows with different editing efficiencies are shown in Table 2. CBE, ABE, and GBE all seem to show preferences for editing at TC or TA motifs with a higher efficiency than other motifs [44,94,99,105], probably due to the deaminase-specific substrate preference. Recent research [109] has tested thousands of target sites and concluded that base editing activity strongly depends on the combination of its position in the target and adjacent base. A preceding T leads to a wider editing window in both CBE and ABE, whereas a preceding G in CBE, and A in ABE, leads to a narrower one, and following T increases C to G editing in CBE. Although the mechanisms behind the variability have not yet been revealed, based on the large target library datasets, the machine learning models, such as FORECasT-BE [109], BE-Hive [110], BE-DICT [111], etc., were trained to predict the editing outcomes of BEs with high accuracy, guiding the applications of current and novel BEs.

### 3.3. Unavailability of Some BEs in Microbes

As reported in previous studies, some BE constructs are unavailable to some microbes, such as *E. coli* [36,62], *Streptomyces* [45,46], *B. subtilis* [55,56], *Psedomonas* [70], *Agrobacterium* [74], and *S. meliloti* [75]. The toxicity of CRISPR/Cas9 has also been reported [112,113]. Although there is no mechanism to explain the unavailability, some solutions can be put forward. Replacing strong promoters with relatively weak promoters, or adding LVA tag to decrease the expression of fusion protein, worked in some cases. The integration of fusion protein into a genome also efficiently worked [57]. Recently, a platform for in vivo rapid investigation of BE components (fusion proteins and sgRNA) in *E. coli* (IRI-CCE) was developed to assess the differential-strength, promoters-driven base conversion [114]. IRI-CCE revealed an appropriate amount of BE expression, which was a crucial factor in achieving high editing efficiency [114], and could be further expanded upon in other microbes.

## 4. Future Scope of BEs in Other Microbes

**Figure 4:**
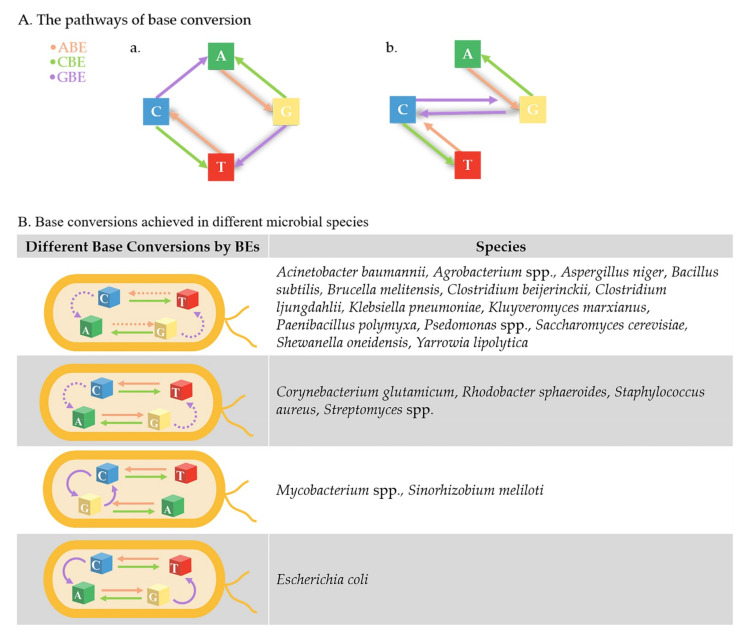
Current development of BEs in different microbial species. (**A**) In theory, any base pair could be converted to any of the others, which only takes three steps at most using the combination of ABE, CBE, and GBE. Firstly, C:G and T:A can be converted to each other by CBE and ABE. Secondly, C:G can be converted to G:C or A:T by GBE. (**a**) If C:G is predominantly converted to A:T by GBE, the most complicated editing step, shaded in grey, is from A:T to C:G, by firstly converting A:T to G:C by ABE, then to T:A by GBE, and finally to C:G by ABE. (**b**) If C:G is predominantly converted to G:C by GBE, the most complicated conversion, shaded in grey, is from A:T to T:A, firstly converting A:T to G:C by ABE, then to C:G by GBE, and finally converting to T:A by CBE. (**B**) The establishment of BEs in different microbes are listed in the table. The achieved base conversions are drawn with a solid line, and base conversions that have not been completed in some microbes are drawn with a dotted line.

Thus far, efficient BE working systems have been established in lots of microbes. In some, base pairs could be converted to any of the others using a combination of CBE, ABE, and GBE. The microbes that have not been fully established by BEs are listed in Figure 4 and will hopefully be explored in the future. We also believe that the nonmodel microbes belonging to the same genera of the universal phylogenetic tree in Figure 2, or that are available for CRISPR/Cas9, hold great hope for the future establishment of BEs. The reason why there has been no huge applications of BEs in other microbes is probably due to the novelty of Bes, since they have just been developed in recent years. The toxicity of fusion protein is another reason, but it can probably be solved by replacing nCas9 with dCas9 and decreasing its expression level as mentioned above. The applications of BEs may also be affected by the polyploidy in some species, as multiplex gene editing is not efficient in some microbes; however, BEs still show powerful potential for precise gene editing and genome-wide engineering in other microbes. The primary reasons are as follows.

Firstly, BEs are more applicable than CRISPR/Cas9 for microbes, especially nonmodel microbes. For those species available for CRISPR/Cas9, BEs are very convenient for plasmid construction and transformation. For those unavailable for CRISPR/Cas9, BEs can still achieve editing with high efficiency since the working process does not depend on DSBs and HR [94,105,106]. The CRISPR/Cas9 and BEs can also complement gene editing. For example, they are designed as a double-check system to increase the editing efficiency of BEs to 99% at some sites [38]. The inactivation of NHEJ genes by BEs facilitate the further application of CRISPR/Cas9 [69]. The insertions of heterologous metabolic pathways into a genome require CRISPR/Cas9 and in situ mutagenesis to increase the industrial production need for BEs [115].

Moreover, the availability of a BE can cover almost the whole genome in microbes. For example, only 24 genes accounting for 0.77% of the genome in *C. glutamicum* [40], and 32 genes in *B. subtilis* [55], are inaccessible for Cas9 variant-BEs by genome-wide analysis. The mutable and knockout targets for CBE account for 99.8% and 96.6%, respectively, in the genome of *R. sphaeroides* [48]. The editable targets account for 99.83% in *Clostridium ljungdahlii* [72], and 96% of editable stop sites are located in the first 80% of the coding region in *S. meliloti* [75]. For *S. aureus*, almost 68.8% of the genes in MRSA252 strain, and 70.36% of the genes in the Newman strain, possess editable stop sites by CBE [79], and 75% ORF can be inactivated in *M. tuberculosis* [79]. Thirdly, BEs can introduce multiplex random mutagenesis in up to five targets in *Streptomyces* [45] and *B. subtilis* [57] for metabolic pathway engineering. The engineering of *B. subtilis* with an enhanced stability to secrete heterologous proteins only takes two rounds of editing to inactivate eight extracellular protease genes [55].

On the one hand, BEs are perfect for precise gene editing, with fewer indels than CRISPR/Cas9. On the other hand, the capability of BEs to produce in situ mutation provides a new method for microbial evolvement. In vivo evolution of proteins was successfully achieved by BEs [57], and was superior to in vitro protein evolution limited by hard heterologous expression. By mutating different sites of genes, the mutant libraries are obtained and can be selected for further analysis. Through this method, mutant stains with improved or decreased performance can be acquired and further used in industrial production and new drug strategies. For example, a *B. bacillus* mutant with an evolved Bacitracin-resistant protein (BceB) can be constructed as a bacitracin-producing cell factory [57]. In situ mutagenesis of a general transcription factor gene *SPT15* gained 36 *S. cerevisiae* mutant strains with sensitive or enhanced stress tolerance, which can be adaptable to various harsh industrial conditions [116]. The mutant libraries also provide large valuable information for future research. Moreover, CBE-mediated multiplex gene editing was developed into a Base Editor-Targeted and Template-free Expression Regulation (BETTER) method in *C. glutamicum* and *B. subtilis*, generating large numbers of genetic combinations of diverse ribosome binding sites, 5′ untranslated regions and promoters to improve xylose catabolism, glycerol catabolism, and lycopene biosynthesis [115]. A BE-mediated in vivo mutagenesis method was further engineered with different systems. Recently, various base deaminases (AID, rAPOBEC1, pmCDA1, and TadA*) were separately fused to T7 RNA polymerase (T7RNAP), which specifically recognized a T7 promoter oriented towards the target sequence, to introduce random mutagenesis as an efficient strategy for continuous in vivo evolution of proteins and metabolic engineering [117,118]. A random base editing (rBE) system was developed by fusing an unspecific single-stranded DNA (ssDNA)-binding protein with rAPOBEC1 in *S. cerevisiae* to achieve genome-scale mutations, and it finally obtained a yeast cell factory resistant to 9% isobutanol [119]. Since the strategies above can be adapted in various organisms, it is possible to look forward to the further evolution of nonmodel microbes.

## 5. Conclusions

In summary, BEs have currently performed efficient editing in 21 microbial genera (Figure 2) and a wide range of nonmodel microbes (Table 2), and they have strong potential to be extended into other species due to their great availability. The applications of BEs in nonmodel microbes are not only limited to precise point mutation, such as gene *inactivation and amino acid substitution, but also genome-wide engineering, such as in vivo* protein and strain evolution. Although the methods have overcome the limitations of CRSIPR/Cas9 in some microbes, their specific obstacles, such as gRNA-independent off-targets, also need to be improved in the future. However, collectively speaking, BEs are expected to be powerful gene engineering tools which speed up the genetic characterizations and metabolic reprogramming in nonmodel microbes.

## Data Availability

Not applicable.
